# Laser weeding: opportunities and challenges for couch grass (*Elymus repens* (L.) Gould) control

**DOI:** 10.1038/s41598-024-61742-8

**Published:** 2024-05-15

**Authors:** Christian Andreasen, Eleni Vlassi, Najmeh Salehan

**Affiliations:** Department of Plant and Environmental Sciences, Faculty of Science, University of Copenhagen, Taastrup, Denmark

**Keywords:** *Agropyrum repens*, Integrated weed management, Perennial weeds, Non-chemical weed control, Site-specific weed management, Thermal weed control, Plant reproduction, Environmental sciences

## Abstract

Laser weeding may contribute to less dependency on herbicides and soil tillage. Several research and commercial projects are underway to develop robots equipped with lasers to control weeds. Artificial intelligence can be used to locate and identify weed plants, and mirrors can be used to direct a laser beam towards the target to kill it with heat. Unlike chemical and mechanical weed control, laser weeding only exposes a tiny part of the field for treatment. Laser weeding leaves behind only ashes from the burned plants and does not disturb the soil. Therefore, it is an eco-friendly method to control weed seedlings. However, perennial weeds regrow from the belowground parts after the laser destroys the aerial shoots. Depletion of the belowground parts for resources might be possible if the laser continuously kills new shoots, but it may require many laser treatments. We studied how laser could be used to destroy the widespread and aggressive perennial weed *Elymus repens* after the rhizomes were cut into fragments. Plants were killed with even small dosages of laser energy and stopped regrowing. Generally, the highest efficacy was achieved when the plants from small rhizomes were treated at the 3-leaf stage.

## Introduction

Extensive use of herbicides has put massive selection pressure for weed resistance to herbicides worldwide^1^ and led to negative side-effects such as pollution of feed, food, and the environment^2–4^. Therefore, many countries and the EU aim to reduce the use of herbicides significantly^5,6^.

Integrated weed management (IWM) is an approach to integrating various weed control methods. IWM can potentially reduce the environmental impact of individual weed management practices and reduce the selection pressure of herbicide resistance^7^. Mechanical weeding is widely used in organic crop production, but there are many other methods which can reduce the selection pressure for herbicide resistance, such as electric weeding^8^, steaming^9^, and flaming^10^. Furthermore, small autonomous weeding robots have been developed to replace tractors and compensate for the lack of agricultural labor power^11,12^.

Autonomous vehicles equipped with lasers to control weeds have gained increasing attention (e.g., https://welaser-project.eu/; https://weedbot.eu/; https://carbonrobotics.com/). Artificial intelligence has enabled rapid and precise identification and location of weeds and crops^13^, and mirrors can guide lasers to target the apical meristem of the weed plants^14^. Plants at the early stage of development can be severely harmed or killed when the apical meristem is exposed to the heat from a laser beam^15,16^. The advantage of using lasers is that only a tiny field area is exposed to the treatments. In contrast, herbicide and mechanical weeding usually expose the whole area to the treatment, which may result in harmful environmental consequences. When a laser beam with a diameter of 2 mm is used to control 75 weeds m^−2^, less than 0.03% of the area is exposed to the treatment. Therefore, laser weeding is the most site-specific achievable weed control method. Laser weeding only leaves behind ashes from the hit plants and does not disturb the soil. Furthermore, lasers are powered by electricity, which can be supplied by batteries and charged from renewable (non-fossil) energy sources, reducing CO_2_ emissions.

If insects or other organisms are placed on the target meristem, or, for example, fly into the laser beam, they will be killed, but the risk is low due to the tiny target area^17^. The earthworm mortality in the soil seems unaffected by the laser beam^17^.

Physical parameters (e.g., beam diameter, laser wavelength, and dose (J))^14^ and biological factors (e.g., plant species, plant size, and developmental stage)^16,18–20^ have importance for the impact of the laser on plants and other organisms. Large plants require higher dosages than small plants to achieve the same effect^20^. Plants that have developed more than one meristem may regrow from lateral meristems when the apical meristem is killed^20^. Consequently, exposing several meristems for the laser beam may be necessary, which complicates and slows down the weeding process. Therefore, laser weeding of weed seedlings should be done when weeds are at the early developmental stages^19,20^.

Small weed seedlings at the cotyledon or 2-leaf-stage are usually killed when they are exposed to a laser dose of ca. 16 J m^−2^, while larger plants may need a larger dose or several treatments as they may regrow^20^. Special challenges are perennial weeds, which can regrow from rhizomes or root parts when the aboveground parts are killed by the laser. Killing rhizomes or roots by continuously killing new shoots with the laser and forcing the belowground part to resprout might be possible. Still, it would require many treatments and be very energy consuming and expensive. However, if the belowground parts are cut into small pieces by soil tillage beforehand, it might be easier to kill the sprouting plants with a laser robot and deplete the belowground parts from resources.

*Elymus repens* is one of the most widespread and troublesome perennial grasses in European agricultural and horticultural fields^21^. It is a highly allelopathic, competitive perennial grass that propagates sexually through seeds and asexually through rhizomes. It is present on all continents except Antarctica but especially in the temperate regions of the world^22,23^. It has been listed as one of the world’s worst weeds^24^. In Northern Europe, *E. repens* is a common and aggressive grass species favored by cereal-dominated crop rotations and nitrogen fertilization^25,26^. *Elymus repens* forms dense patches that tend to exclude most other species. Even low levels of *E. repens* growth often lead to significant crop yield reductions due to direct competition for nitrogen and moisture. However, repeated weed surveys in Northern Europe have shown that the extensive herbicide use in the region, particularly glyphosate, has led to a decline in *E. repens* frequency^27,28^. However, new restrictions on glyphosate use in Europe call for alternative methods to control *E. repens.*

This project aims to investigate how the *E. repens*, established from small pieces of rhizomes, can be killed by exposing the plants to increasing dosages of laser energy up to a dose of 15.9 J mm^−2^. We used a fibre laser with a wavelength of 2 µm and a 2 mm diameter. The water inside the target absorbs the 2 µm wavelength. A thulium-doped 2 µm fibre laser has been installed in the autonomous vehicle for laser weeding developed in the EU project WeLASER (https://welaser-project.eu/). A laser energy dose below 20 J mm^−2^ may be used to control small weed plants in crop fields^15,18,19^. We hypothesized that depending on plant size, it would be possible to kill and prevent regrowth from small belowground parts after two laser treatments.

## Materials and methods

### Laser equipment

A thulium-doped 50 W fibre laser with a wavelength of 2 µm manufactured by Futonics Laser GmbH, Katlenburg-Lindau, Germany, was used. The laser had a collimated beam (Ø: 2 mm). Plants were irradiated in a steel box (68 cm × 68 cm × 68 cm) with a door with a metal interlock^29^. The door locked automatically when the laser was activated to avoid exposing users to reflecting laser beams. The laser dose was determined and activated from a computer. Plants were placed approximately 30–35 cm from the laser head and exposed to increasing dosages of laser energy up to 15.9 J mm^−2^ from an angle of 45°. The laser was pointed directly towards the plant approximately 1 cm above the ground to cut the plant from one side. The dose was determined by the time (s) the target was exposed to the irradiation, and the energy consumption per mm^2^ was calculated using Eq. 1.

1 $${\text{Dose }}\left( {{\text{J mm}}^{{ - {2}}} } \right) \, = \, \left( {{5}0{\text{ W }} \times {\text{ s}}} \right) \, / \, \left( {{22}/{7 } \times { 1}^{{2}} {\text{mm}}^{{2}} } \right)$$

### Plants

**Figure 1:**
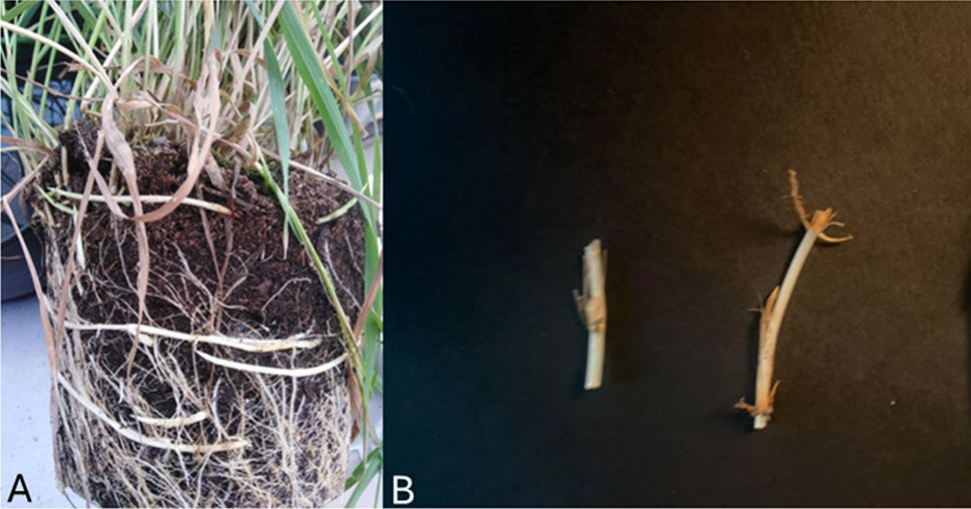
(**A**) *Elymus repens* growing in a pot to produce rhizomes. (**B**) Cut pieces of rhizomes with one and two nodes used in the experiments.

Plants of *Elymus repens* (L.) Gould were grown in plastic pots outside a greenhouse for about a year (Fig. 1A). The rhizomes were dug up and cut into pieces with either one or two nodes and used in the experiments (Fig. 1B). Each piece of rhizome was placed in a plastic pot (height: 7 cm; Ø: 10 cm) containing a sphagnum soil (Pindstrup Ready Mix 2 (https://www.pindstrup.dk/professionel/product-details/pindstrup-f%C3%A6rdigblanding-2)) and covered with 1 cm of the soil. The pots with the same rhizome type (with one or two nodes) were placed randomly in plastic trays (58 cm × 30 cm) with holes in the bottom to facilitate irrigation. The pots were kept in a greenhouse until the rhizomes with one node had established the desired growth stages (1-leaf, 2-leaf, or 3-leaf stage). Plants from rhizomes with two nodes were kept in the greenhouse until they established 1 or 2 shoots with one, two, or three leaves. The pots were watered daily by applying water through the bottom of the tray during the whole experimental period to ensure that water was not a limiting growth factor. After approximately 3–5 weeks, depending on the temperature, the rhizomes had established plants on the desired growth stage and were moved to the laser cabinet and irradiated. All experiments were conducted between February and December 2023. Experimental research on plants (either cultivated or wild), including the collection of plant material was carried out in compliance with relevant institutional, national, and international guidelines and legislation.

### Lasering and subsequent plant growth

Experiment 1: Plants established from rhizomes with one node with 1, 2, or 3 leaves, respectively, were exposed to one of the following doses: 0 s, 0.05 s, 0.1 s, 0.3 s, 0.5 s, 0.8 s, and 1 s corresponding to 0 J mm^−2^, 0.8 J mm^−2^, 1.6 J mm^−2^, 4.8 J mm^−2^, 8.0 J mm^−2^, 12.7 J mm^−2^, and 15.9 J mm^−2^(Experiment 1). We used 3–6 replicates for each developmental stage. After the irradiation, the plants were placed randomly on a shelf in a climate cabinet at 21 °C with 12 h of light and 12 h of darkness. Twenty-one days later, the plants were transferred to the laser cabinet and again irradiated with the same dose using the same method. After the second irradiation, the plants were placed randomly on a shelf in the climate cabinet. Approximately 21 days after (Table 1), the plants were cut just above the ground, and the fresh weight of the leaves was recorded. The rhizomes were thoroughly rinsed with tap water, dried with a dry piece of paper, and weighed.

**Table 1:** Dates the lasering and harvest were conducted.

	Stage	1st treatment	2nd treatment	Harvest
Experiment 1: Rhizomes with one node				
Run 1	1-leaf	23-02-2023	16-03-2023	06-04-2023
	2-leaf	29-06-2023	20-07-2023	10-08-2023
	3-leaf	25-08-2023	15-09-2023	06-10-2023
Run 2	1-leaf	02-06-2023	23-06-2023	14-07-2023
	2-leaf	15-08-2023	05-09-2023	26-09-2023
	3-leaf	27-10-2023	17-11-2023	08-12-2023
Experiment 2: Rhizomes with two nodes and one shoot				
Run 1	1-leaf	16-05-2023	06-06-2023	27-06-2023
	2-leaf	21-04-2023	12-05-2023	02-06-2023
	3-leaf	21-04-2023	12-05-2023	02-06-2023
Run 2	1-leaf	10-08-2023	31-08-2023	21-09-2023
	2-leaf	15-08-2023	05-09-2023	26-09-2023
	3-leaf	25-08-2023	15-09-2023	06-10-2023
Experiment 3. Rhizomes with two nodes and two shoots				
Run 1	1-leaf	12-06-2023	03-07-2023	25-07-2023
	2-leaf	12-06-2023	03-07-2023	25-07-2023
	3-leaf	30-10-2023	20-11-2023	11-12-2023
Run. 2	1-leaf	10-08-2023	31-08-2023	21-09-2023
	2-leaf	30-10-2023	20-11-2023	11-12-2023
	3-leaf	Lacking		

Experiment 2 and 3. Three to six replicates of plants established from rhizomes with one or two nodes (Exp. 2 and Exp. 3, respectively) were irradiated when they had 1 or 2 shoots with one, two, or three leaves, respectively, using the same dosages and subsequently treated as plants established from rhizomes with one node.

Thus, each dose–response experiment consisted of 3(− 6) pots × 7 doses and was repeated once for all combinations (Run 1 & 2), except the experiment with two nodes, two shoots and three leaves, which was only done once due to lack of plants. Six replicates were used in one experiment with rhizomes with two nodes and one leaf, (6 pots × 7 doses = 42 pots) (Table 1). In total, 399 plants were irradiated (three replicates (6 for two experiments) × 7 doses × 2 types of nodes (× 2 for the two nodes category: one or two shoots) × 3 developmental stages × 2 experiments − (3 × 7 plants)).

### Statistical analyses

All data sets were individually analyzed, as the experiments were considered independent, as the desired growth stages were established at different times with varying temperature and light conditions in the greenhouse (Table 1). The physiological stage of the rhizomes (their ability to sprout and grow) could be affected by the season. Rhizome fragments taken late in the season may be more or less vigorous than fragments taken early in the season. The response, *y*, was described by a log-logistic dose–response curve using the statistical software R version 4.2.0^30^ with the add-on drc package (version 4.2.3). A three-parameter model was used to describe the data:

2 $$y=\frac{d}{1+{\text{exp}}[b\left({\text{log}}(x\right)-{\text{log}}(e))]}$$

*y* is the biomass 21 days after the treatment, *d* is a parameter close to the untreated control (upper limit = maximum biomass). The parameter *b* is proportional to the slope of the curve at dose *e,* which is the effective dose that reduces the biomass by 50% (ED_50_). The effective doses ED_10,_ ED_50,_ and ED_90_, resulting in a 10, 50, or 90 per cent biomass reduction, respectively, were estimated. All data are available in Supplementary Material.

## Result

### Experiment 1: Plant established from one node

**Figure 2:**
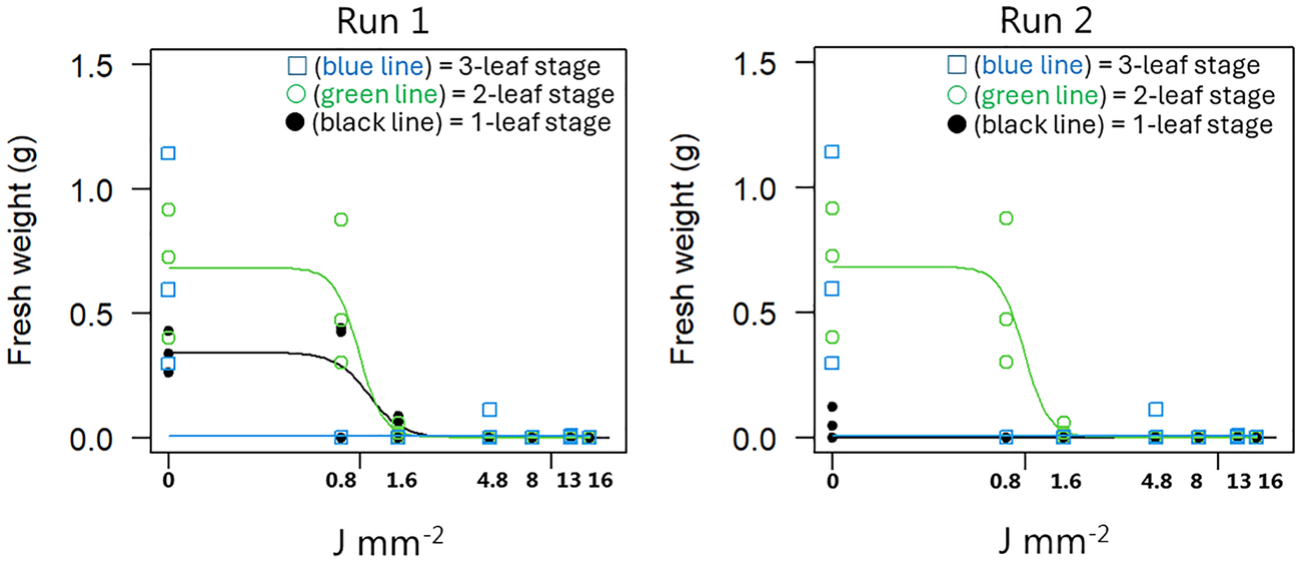
Aboveground fresh weight of *Elymus repens* plants established from rhizome fragments with only one node 21 days after the second laser treatment. Developmental stages when plants were treated with laser: ● (black line) = 1-leaf stage;  (green line) = 2-leaf stage;  (blue line) = 3-leaf stage.

*Elymus repens* plants established from rhizomes with one node were all susceptible to laser irradiation with doses ≥ 1.6 J mm^−2^. The dose of 1.6 J mm^−2^ killed almost all plants at all three developmental stages (Fig. 2). Plants irradiated on the 1- and 2-leaf stage with a dose of 0.8 J mm^−2^ were able to reestablish and produce just as much biomass as the controls 21 days after the second treatment in Run 1 (Fig. 2). The same happened in Run 2 for the 2-leaf stage, but the biomass production of plants in the 1-leaf stage in Run 2 did not grow much (Fig. 2; Table 1). In both experiments, plants at the 3-leaf stage were all killed at all laser dosages.

### Experiment 2: plant established from two nodes with one shoot

**Figure 3:**
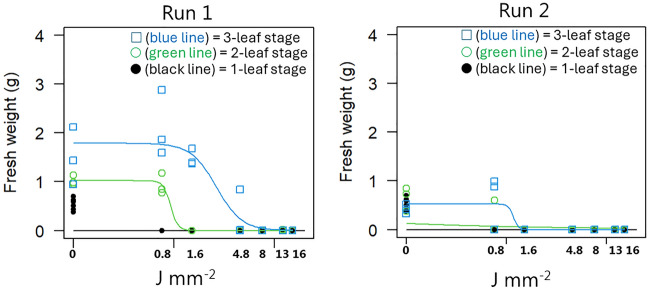
Aboveground fresh weight of *Elymus repens* plants with one shoot established from rhizome fragments with two nodes 21 days after the second laser treatment. Developmental stages when plants were treated with laser: ● (black line) = 1-leaf stage;  (green line) = 2-leaf stage;  (blue line) = 3-leaf stage.

When rhizome fragments with two nodes sprouted with only one shoot (Fig. 3), a dose of 8 J mm^−2^ was necessary to stop regrowth in Run 1, while a dose of only 1.6 J mm^−2^ was enough in Exp. 2. Generally, the biomass production was considerably larger in Run 1 than in Run 2 due to better growth conditions in the summertime. At doses lower than 1.6 J mm^−2^, plants irradiated at the 3-leaf stage produced more biomass than plants treated at the 2-leaf stage, which contrasts with plants sprouting from rhizomes with one node, where the 3-leaf stage was the most susceptible.

### Experiment 3. plant established from two nodes with two shoots

**Figure 4:**
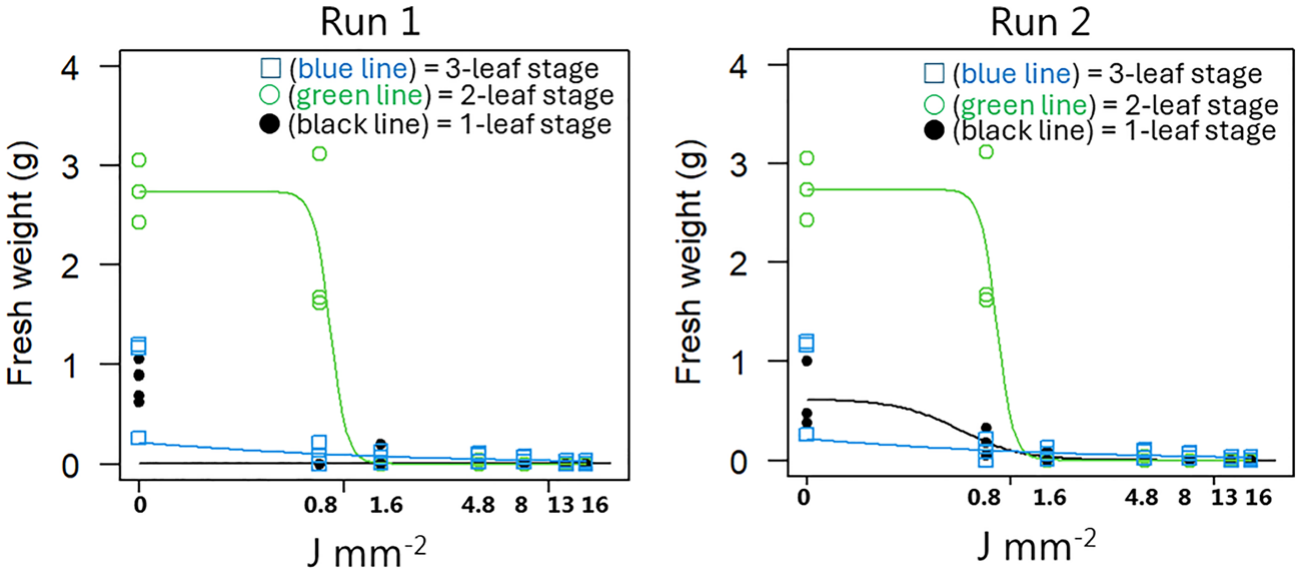
Aboveground fresh weight of *Elymus repens* plants with two shoots established from rhizome fragments with two nodes 21 days after the second laser treatment. Developmental stages when plants were treated with laser: ● (black line) = 1-leaf stage;  (green line) = 2-leaf stage;  (blue line) = 3-leaf stage.

When rhizome fragments with two nodes sprouted with two shoots (Fig. 4), a dose of 1.6 J mm^−2^ was enough to stop biomass production for all leaf stages in both Runs. Lower dosages did not prevent plants treated at the 2-leaf stage to reestablish and produce almost as much biomass as the controls. However, plants treated on the 1- and 3-leaf stage failed to produce biomass even at the lowest dose (0.8 J mm^−2^). The lack of doses below 0.8 J mm^−2^ made the estimating of the dose–response curve imprecise. The dose range was chosen because we expected that it was necessary to use much higher energy levels to kill the plants.

## Discussion

Even small doses had a good effect on small fragments of rhizomes, indicating that laser could be suitable in integrated weed management strategies in areas where herbicide application is not accepted (e.g., in organic agriculture, areas reserved for extraction of drinking water) or where the selection pressure for glyphosate and other herbicide resistances should be avoided or reduced. The study focused on small rhizome fragments, but even after several soil tillage’s, larger rhizomes with more than two nodes are likely present in the field. Therefore, there is also a need to study the laser effects on larger fragments of rhizomes and investigate how many laser treatments are necessary to destroy the above-ground biomass to deplete the stored resources in the rhizomes. This can also be done by intensive soil tillage repeated over a long period (Håkansson^31^), but that is not desirable. If laser weeding could replace some soil tillage treatments, it would benefit the environment because the laser only affects a small area corresponding to the number of aerial shoots of perennial weed species and germinating plants.

When *E. repens* has established 3–4 leaves, the regenerative structures have their minimum dry weight and lowest tillage tolerance^31^. We also found the best laser effect when the rhizomes with one node had three leaves (Fig. 2) and rhizomes with two nodes and two aerial sprouts had three leaves (Fig. 4). However, when rhizomes with two nodes only had one aerial spout, the susceptibility to laser decreases with an increasing number of leaves, which contrasts with the expectation (Fig. 3). Remarkably, the laser efficacy was higher when both rhizome types had one leaf than when they had two. We did not study whether the plants became infected with fungi after the treatment. However, seeds exposed to increasing dosages of laser have shown increasingly severe fungal infection^29^, and an early infection may have more severe consequences for the plant than later contamination. That may explain why the 1-leaf stage was less laser-resistant than the 2-leaf stage.

The aerial shoots and rhizomes of *E. repens* constitute a sympodial system. Rhizome growth is renewed annually from axillary buds at the base of the aerial shoots. Axillary buds along the rhizomes are mainly dormant due to the solid apical dominance exerted by the terminal bud. Aerial shoots are primarily formed at the end of the growing season when a rhizome tip becomes erect; they are also formed from both axillary and terminal buds when a rhizome becomes detached from the parent plant. Axillary buds on a rhizome are released from dormancy when the rhizome apex is removed or when the rhizome is severed from the parent plant. Isolated rhizome segments exhibit a polarity such that buds toward the apical end develop into aerial shoots, and those toward the base become rhizomes or usually remain dormant^32^. However, in our study, many rhizomes with two nodes established aerial shoots from both nodes. In experiment 2, Run 2, (Fig. 3) the biomass production was significantly smaller than in Run 1, because of less vigorous rhizome fragments in the autumn. Adventitious roots form at nodes of the rhizome. Roots are short in length relative to other grasses. Rhizomes typically grow within the topsoil layer; the great majority of them mainly in the upper 10 cm soil layer in undisturbed growth^31^, and therefore, rhizomes can easily be divided into small fragments with a tine harrow and afterwards controlled with a laser instead of using several passes of harrowing. However, fragmentation of rhizomes can lead to further spreading of plants in the field if the sprouting fragments are not controlled, for example, by laser.

### Estimations of models

Several of the models did not fit data well due to the large variation in data and especially the lack of observations at low doses (< 1.6 J M^−2^) resulting in poor estimations of model parameters for some of the S-shaped curves (Tables 2). Observations around the ED50-value are essential to get a good fit. Including doses between 0 and 1.6 J M^−2^ could have improved the estimation of the dose–response relationship significantly.

**Table 2:** Estimated model parameters (see Eq. 2). The ED_10,_ ED_50,_ and ED_90_ values are the effective doses resulting in a 10, 50, or 90 per cent biomass reduction.

Run	Leaf stage	Parameter	One node, one shoot		Two nodes, one shoot		Two nodes, two shoots	
			Value	SE	Value	SE	Value	SE
1	1-leaf	*b*	5.0	2.6	− 0.08	0.51	− 3 × 10^–2^	5 × 10^–2^
		*d*	0.34	0.05	0.69	10.2	1.11	10
		*e* (ED50)	1.11	0.22	1 × 10^39^	10	4.2	10
		ED10	0.72	0.24	6 × 10^26^	1 × 10^29^	6 × 10^–54^	2 × 10^57^
		ED90	1.72	0.47	4 × 10^51^	7 × 10^53^	3 × 10^–03^	Inf^a^
1	2-leaf	*b*	6.8	4.6	11.8	15.6	10.8	31.8
		*d*	0.68	0.08	1.020	0.04	2.7	0.17
		*e* (ED50)	1.0	0.17	0.93	0.24	0.85	0.30
		ED10	0.7	0.1	0.77	0.05	0.70	0.18
		ED90	1.36	0.48	1.12	0.56	1.0	1.0
1	3-leaf	*b*	− 0.08	0.23	3.42	1.23	0.46	0.67
		*d*	1.2	10	1.79	0.17	0.87	0.10
		*e* (ED50)	6 × 10^30^	10	2.65	0.72	0.01	0.07
		ED10	5 × 10^19^	3 × 10^21^	1.40	0.53	7 × 10^–5^	0.001
		ED90	8 × 10^41^	5 × 10^43^	2.65	0.72	1.1	2
2	1-leaf	*b*	5.0	2.7	-0.08	0.51	2.8	3.2
		*d*	0.34	0.05	0.69	10.2	0.6	0.07
		*e* (ED50)	1.11	0.22	1.5 × 10^39^	10	0.57	0.25
		ED10	0.71	0.24	5.6 × 10^26^	1 × 10^29^	0.26	0.04
		ED90	1.72	0.47	3.9 × 10^51^	7 × 10^53^	1.2	0.7
2	2-leaf	*b*	6.81	4.64	0.35	0.16	10.8	31.8
		*d*	0.68	0.07	0.66	0.09	2.7	0.2
		*e* (ED50)	0.99	0.17	0.002	0.004	0.86	0.3
		ED10	0.7	0.12	3 × 10^–6^	1 × 10^–5^	0.7	0.18
		ED90	1.36	0.35	0.84	1.76	1.1	1.0
2	3-leaf	*b*	− 0.08	− 0.02	17.8	40.0	0.46	0.67
		*d*	1.2	10	0.52	0.08	0.88	0.12
		*e* (ED50)	6 × 10^30^	10	1.18	0.85	0.01	0.07
		ED10	5 × 10^19^	3 × 10^21^	1.04	0.85	7 × 10^–5^	1 × 10^–03^
		ED90	9 × 10^41^	5 × 10^43^	1.18	0.85	1.1	2.0

^a^Inf = infinity.

SE = Standard Error.

### Comparing laser weeding with herbicide application

Laser weeding seems to be a promising tool to supplement herbicides. Laser weeding only leaves the ash from the burned plant tissue in the field after the treatment, mainly consisting of phosphorous and calcium, essential plant nutrition for the crop. Herbicides may evaporate or leach to surface and groundwater and may expose the environment to short or long-term unwanted side effects and may contaminate food and feed^33–35^.

Glyphosate application has, with great success, reduced the spread and occurrence of *E. repens* in Europe, mainly by spraying in cereals before harvest to kill *E. repens* and other weeds and dry the crops^27,28^. After intense debate, The European Commission has decided to renew the license for glyphosate, approving its use in European Union countries for ten more years (until 2033). Based on comprehensive safety assessments carried out by the European Food Safety Authority (EFSA) and the European Chemicals Agency (ECHA), the European Commission has renewed the license with certain new conditions and restrictions. These include a ban on the use of the chemical to dry crops before harvest and “the need for certain measures to protect non-target organisms”. Governments can still restrict the use of glyphosate in their own countries if they consider the risks too high, particularly regarding the need to protect biodiversity^36^. These restrictions may reduce the possibility of controlling *E. repens* with glyphosate leading to increasing infestations and yield losses. Therefore, there is an increasing demand to develop new methods that can contribute to its control. It is also essential to be prepared if glyphosate application will be forbidden after 2033, but 10 years is a realistic period to develop, produce, and market efficient laser weeding robots. Some are already on the market (see https://carbonrobotics.com/; https://weedbot.eu/weedbot-products/).

### Comparing laser weeding with soil tillage

Repeated soil tillage has several negative impacts on the environment. Soil tillage harms shallow living earthworms and other essential soil organisms when harrowing tines or other implements pass through the topsoil^37^. In contrast, earthworm mortality seems unaffected by laser weeding^17^. Soil tillage also harms beneficial organisms on the soil surface, like parasitic wasps, ladybugs, spiders, and predatory beetles^38–41^. In contrast, the probability of hitting insects with the laser is small because of the tiny laser target area. Therefore, laser weeding is more eco-friendly than many other weed-control methods. Furthermore, soil tillage may dry out soils with limited moisture content, causing erosion, release and leaching of plant nutrients, and unnecessary degradation of organic matter, resulting in increased CO_2_ emissions^42,43^.

Replacing mechanical weeding with a laser would be possible in fields with only annual weeds if they are irradiated at the cotyledon stage or 2–4 leaf stages^19,20^. Still, it would require too much energy to control perennial weeds with widespread belowground biomass that continuously produces new aerial shoots after the laser has killed the aboveground parts. Therefore, we recommend combining mechanical weeding with laser, cutting belowground parts into small fragments, and thereby easing the depletion of stored resources in the fragments to kill them, as shown in our experiments. That would reduce the need for more tillage passes. The effect of laser on plants established from larger rhizome fragments and with more nodes than we used must also be studied as larger fragments commonly occur even after several mechanical weeding passes.

### Laser capacity

If a dose of 12.7 J m^−2^ is necessary to kill *E. repens* spouts, it would take 0.8 s plant^−1^ with a 50 W fibre laser (Eq. 1). With a dense stand of *E. repens*, for example 75 spouts m^−2^, it would take one minute to control 1 m^2^, resulting in slow driving speed. Therefore, it is necessary to use more powerful lasers in autonomous laser vehicles. In the WeLASER project (https://welaser-project.eu/), the intention was to install 500 W fibre lasers, resulting in a ten times higher weeding capacity^44^.

### Laser safety

In the experiments, a collimated beam was used. A collimated laser beam is not appropriate for practical field work. If a collimated beam hits a piece of metal, glass, or stone, it may be reflected and escape the target area, and animals (hares, mice, birds, dogs, etc.) and humans may be blinded or burned by the concentrated beam. In laser weeding robots, the beams should only be concentrated on the target area of the plant. If the laser accidentally is reflected, it will be spread in a cone, and the risk of harming humans, animals or other plants would be significantly reduced due to a much lower dose per area. Only insects sitting precisely in the focus point would be exposed to the dose determined for the weed. The further away from the focus point, the smaller the dose an organism would receive, and the less harmful the irradiation would be.

## Conclusion

The experiments showed that *E. repens* plants established from small rhizome fragments with one or two nodes could be harmed or killed with laser energy doses less than what is expected to be used to control weed seedlings (ca. 20 J mm^−2^) after two treatments. When plants were established from small rhizomes with one node, they were most susceptible at the 3-leaf stage, followed by the 1-leaf stage and 2-leaf stage. When rhizomes with two nodes produced one shoot, the susceptibility decreased with the number of produced leaves. If rhizomes with two nodes produced two shoots, the 2-leaf stage was the most laser-resistant stage. The experiments showed that it is possible to kill *E. repens* plants with small laser doses (in many cases, with less than 1.6 J mm^−2^). Cutting *E. repens* into smaller fragments and subsequently killing aerial shoots with a laser seems to be a promising method to control *E. repens* and reduce the impact of weed control on the environment. The next step will be to study the effect of laser on larger rhizome fragments and under field conditions where the weed plants may be exposed to drought and flooding, and are placed in various depths, which may influence the survival rate significantly.

### Supplementary Information

Supplementary Information.

## Data Availability

All data generated or analyzed during this study are included in this published article and its supplementary information file.
